# Prehospital emergency care patient satisfaction scale [PECPSS] for care provided by emergency medical teams: Scale development and validation

**DOI:** 10.3934/publichealth.2023011

**Published:** 2023-03-01

**Authors:** Junpei Haruna, Nobuyasu Hayasaka, Yukiko Taguchi, Saori Muranaka, Sachi Niiyama, Hirotoshi Inamura, Shuji Uemura, Keigo Sawamoto, Hirotoshi Mizuno, Nobuaki Himuro, Eichi Narimatsu

**Affiliations:** 1 Department of Intensive Care Medicine, School of Medicine, Sapporo Medical University, South-1, West-16, Chuo-ku, Sapporo, Hokkaido, 060–8543, Japan; 2 Emergency Sector of Ebetsu City Fire Department, Yoyogicho, 80–8, Nopporo, Hokkaido, 069–0817, Japan; 3 Department of Nursing, School of Health Sciences, Sapporo Medical University, South-1, West-17, Chuo-ku, Sapporo, Hokkaido, 060–8556, Japan; 4 Department of Advanced Critical Care and Emergency Center, Sapporo Medical University Hospital, South-1, West-16, Chuo-ku, Sapporo, Hokkaido, 060–8543, Japan; 5 Department of Pharmacy, Sapporo Medical University Hospital, South-1, West-16, Chuo-ku, Sapporo, Hokkaido, 060–8543, Japan; 6 Department of Emergency Medicine, Sapporo Medical University School of Medicine, South 1, West 16, Chuo-ku, Sapporo, Hokkaido, 060–8543, Japan; 7 Department of Public Health, School of Medicine, Sapporo Medical University, Sapporo, Japan; 8 Department of Emergency Medical Services, Life Flight and Disaster medicine, Sapporo Medical University, South 1, West 16, Chuo-ku, Sapporo, Hokkaido, 060–8543, Japan

**Keywords:** patient satisfaction scale, prehospital care, emergency medical technicians, scale development, scale validation, exploratory factor analysis, ambulance care

## Abstract

The purpose of this study was to develop and validate an emergency medical technician (EMT) care patient satisfaction scale to measure patient satisfaction with prehospital emergency care. To date, patient satisfaction surveys of EMTs have been performed subjectively, e using each facility's questionnaire, without the use of a validated patient satisfaction scale. However, no specific scale has been devised to assess patient satisfaction with EMTs. The study population comprised patients who used an ambulance between November 2020 and May 2021 (N = 202). A survey instrument was administered to participants who provided informed consent. In the process of validating the patient satisfaction scale, an exploratory factor analysis (EFA) of construct validity was performed. The results of the EFA showed a factor structure consisting of five factors: “teamwork”, “explanation and communication”, “physical treatment and psychological support”, “quickness of transport”, and “environment in the ambulance”. In addition, domain and summary scores showed good internal reliability (Cronbach's range = 0.82–0.94). The patient satisfaction scale developed in this study was designed and validated considering the role of EMTs and patients' needs for prehospital care. This scale may be useful in the development of assessments and interventions to improve patient satisfaction with EMTs.

## 1. Introduction

Patient response to health care services is among the best sources for obtaining information regarding health care quality [1]. In particular, it is an important indicator for evaluating medical services from the patient's perspective and for improving medical care and is one of the most valid indicators commonly used [2],[3]. Moreover, the importance of patient satisfaction is further underscored by evidence confirming that when it is met, patients are more likely to benefit from health care and experience improved quality of life [4]–[6]. The medical field is divided into specialized areas, each with its own distinct treatment and care approaches. In measuring patient satisfaction, the development of surveys for each specialty has been reported to be helpful in addressing specific issues [7].

Emergency medical services (EMS) play a critical role in providing care to patients in prehospital settings worldwide. The EMS field evolved in the 1960s due to the occurrence of traffic trauma and has been advancing since [8]. Presently, the scope of prehospital emergency care is no longer limited to traffic trauma [9]. In addition, the role of emergency medical technicians (EMTs) has diversified due to changes in the structure of diseases and populations, for example, cardiac disease and acute exacerbations of chronic diseases [10]. Therefore, EMS is a common entry point toward a continuum of health care, and the presence of EMTs is critical to provide the necessary medical care in a prehospital setting.

Several reports have been published on patient satisfaction with EMS in prehospital care [11],[12]. However, these reports used satisfaction scales originally developed by the researchers, and the validity and reliability of the scales have not been verified. Although the use of objectively validated and reliable scales is recommended to measure patient satisfaction [13],[14], to our knowledge, no patient satisfaction scale for activities has been reported for prehospital EMTs. The current study aimed to fill this research gap by developing a patient satisfaction survey for EMTs who provide emergency care.

## 2. Materials and methods

### 2.1. Study design

The key objectives of our study, which informed our research design, were as follows: 1) develop items to measure patient satisfaction with EMT services, 2) examine their content validity, 3) select the appropriate items, and 4) examine their construct validity.

#### 2.1.1. Development of items to measure patient satisfaction with EMT services

In this phase, we considered the inclusion of various items according to the Consumer Emergency Care Satisfaction Scale (CECSS) [15] and the Patient Satisfaction Questioner-18 (PSQ-18) [2]. The items for the scale were developed through three consensus meetings held among the researchers, with inputs from experts in instrument development. The study team consisted of nine professionals: two lead EMTs, two critical care nurses, one certified emergency nurse, one emergency nurse, and three emergency physicians [16]. The first version of the questionnaire was based on the five dimensions of satisfactory service, with 52 items distributed among eight components. In addition, we searched and reviewed databases in this field, including CINAHL, PubMed, and Medline, using the keywords “EMT” and “patient satisfaction”. Consequently, 12 items were extracted and 64 items were created.

#### 2.1.2. Content validation

Previous studies have reported that the process of content validation should reflect the opinions of both the patients involved as well as the experts [17]. Content validation of the first version of the questionnaire was conducted by a panel of 30 individuals: 10 healthy people who had used an ambulance, 10 EMTs who had worked for more than 10 years, and 10 faculty members from universities that train EMTs [18]. A questionnaire was sent to them, asking if the survey items were valid. The questionnaire items were assessed on a 4-point Likert scale ranging from 1 (“not at all important”) to 4 (“very important”). Questions were also asked regarding repetitiveness, difficulty in understanding, and ease of answering questions. Following the method proposed by Davis, the item-level content validity index (I-CVI) was calculated by dividing the number of experts who rated each item as 3 or 4 by the total number of experts [19]. Moreover, items with an I-CVI lower than 0.78 were eliminated [20]. The final version of the questionnaire was derived after content validation analysis and the elimination of one item based on the results from various consensus meetings between the research team and expert advisors. As a result, the prehospital emergency care patient satisfaction scale (PECPSS), from 64 items in the first version, was reduced to 32 items in the final version.

#### 2.1.3. Participants

Japan's emergency medical care system is classified as follows: primary emergency facilities, mainly providing outpatient services; secondary emergency facilities, predominantly treating severely ill patients who require hospitalization; and emergency medical centers, treating severely ill patients who require advanced treatment [9]. Patients who cannot visit the hospital independently are transported to an emergency hospital by ambulance, which is requested by either the patients or their family members. EMTs are affiliated with each municipality and are responsible for driving ambulances, providing first aid to patients in the ambulances, and transporting patients to emergency facilities. In this study, fire departments in the Hokkaido region of Japan were asked to cooperate in the survey. Patients who used an ambulance and met the following criteria were given a survey form by hand after explaining the study to them and asked to return it within one month.

- Must be at least 20 years of age at the time of application

- Able to provide consent

- Possess the cognitive and physical ability to complete the self-administered questionnaire without a proxy

Participants comprised patients who used ambulances in one region of Japan between November 2020 and May 2021 and met the above criteria, and they were given the questionnaires; the research collaborator, an EMT, asked each respondent to complete the same. The sample size was targeted to be at least 100 individuals, based on the COSMIN checklist [21], which is a guideline for scale development.

#### 2.1.4. Survey components

The survey consists of four components: 1) a questionnaire on personal characteristics; 2) questions on the number of times and the time of day when an ambulance was used; 3) the EQ-5D-5L [22],[23], which consists of six items to confirm construct validity, and items measuring good reception of the ambulance crew, the intensity of distress in the ambulance, and trust in the EMT, respectively, as measured with a visual analog scale (VAS); and 4) a satisfaction survey regarding the EMTs' service.

#### 2.1.5. Instruments

The EQ-5D-5L is a validated and standardized measure of health-related quality of life [22],[23], with a Japanese version available [24]. The EQ-5D-5L consists of five dimensions: mobility, self-care, usual activities, pain/discomfort, and anxiety/depression. Each dimension has five levels: no problem, mild problem, moderate problem, severe problem, and extreme problem. The EQ-5D-5L also uses a VAS from 0 to 100, representing the worst imaginable health and best imaginable health, respectively. Given that satisfaction with treatment is related to the quality of life, we hypothesized that the PECPSS would be more correlated with quality of life and chose to measure the quality of life using the EQ-5D-5L [25].

To determine the construct validity of the patient satisfaction scale for EMTs, we first reviewed the information available from previous studies [26]–[29]. Second, we extracted factors related to patient satisfaction with EMTs. Third, based on the factors extracted in the second step, we aimed to verify the construct validity of three questions (on EMTs' hospitality, distress during transport, and confidence in the EMTs) formulated based on our results, by interviewing six EMTs. Each question was measured using the VAS, with 100 representing “strongly agree” and 0 representing “disagree”.

For each of the patient satisfaction surveys for EMT services, respondents rated their level of agreement on a standard 5-point Likert scale (1 = “strongly disagree”, 2 = “somewhat disagree”, 3 = “neither agree nor disagree”, 4 = “somewhat agree”, 5 = “strongly agree”).

### 2.2. Statistical analysis

Descriptive statistics were derived for the analysis. Categorical data were expressed as numbers and percentages. Some of the survey items were considered for possible exclusion if the mean score for each item was 4.5, 1.5, or lower. In addition, any item with a correlation coefficient of 0.8 or higher was excluded [30].

Exploratory factor analysis (EFA) with Promax rotation and maximum likelihood methods was used to determine the number and type of factors from the 36 survey items. EFA was conducted on the complete data for all 36 items at baseline. Factor solutions with EFA were based on the size of the factor loadings for each item. Items with factor loadings less than 0.35 were excluded based on standard psychometric criteria. Researchers assessed whether the removal or retention of specific items was meaningful in assessing patient satisfaction.

Based on the results of the EFA, each factor representing various aspects of patient satisfaction with EMT services was categorized and named. Internal consistency reliability of the PECPSS was assessed using Cronbach alpha. Reliability estimates should exceed 0.70 (0.7 ≤ alpha < 0.8 is acceptable, 0.8 ≤ alpha < 0.9 is good, 0.9 ≤ alpha is excellent) [31],[32]. Construct validity was assessed using domain and PECPSS summary scores obtained through the EFA and four validated questionnaires: the EQ-5D-5L (using the VAS), anxiety on the EQ-5D-5L, good hospitality received from EMTs (VAS), degree of distress during transport (VAS), and degree of confidence in EMTs (VAS) were assessed using the Pearson correlation of trust in EMTs (VAS). We hypothesized that higher patient QOL would be positively correlated with higher summary scores of patient satisfaction and that the intensity of patient anxiety would be negatively correlated with summary scores of patient satisfaction. It was also hypothesized that higher summary scores for hospitality and trust toward EMS personnel would correlate with higher patient satisfaction, whereas the degree of distress during transport would not correlate with the summary score for patient satisfaction.

Only questionnaires with complete data were included in the analysis, and missing data were not imputed. Statistical significance was set at p ≤ 0.05 (two-tailed). Statistical analyses were performed using SPSS Statistics version 27 (IBM Corp., Armonk, NY, USA).

### 2.3. Ethical considerations

The protocol for this research project was approved by the Ethics Committee of Sapporo Medical University (approval number: 1–2–51) and conforms to the provisions of the Declaration of Helsinki. Informed consent was obtained from all respondents. Participants were informed of the purpose and duration of the study and their participation was voluntary. Consent was obtained from respondents by their checking the box on the cover page of the questionnaire form indicating that they understood the study and agreed to participate, in accordance with IRB recommendations.

## 3. Results

A total of 210 respondent surveys were included in the final analysis after excluding eight surveys with missing data. Several items of the questionnaire contained missing data, and all of them were missing at random. The response rate was 40.4%. The survey respondents' characteristics are presented in Table 1. Of the total, 101 (50.0%) were male, with an average age of 68.1 years; 64 patients (31.7%) were employed, and 139 patients (63.0%) had underlying diseases. The most common time of transport was 6:00 to 12:00 for 63 patients (31.2%), followed by 18:00 to 00:00 for 55 patients (27.2%).

**Table 1.** Participant sociodemographic and clinical data (N = 202).

Characteristic	
Age means (SD)	68.1 (15.9)
Gender
Male, n (%)	101 (50.0)
Female, n (%)	101 (50.0)
Employment situation
Unemployed, n (%)	64 (31.7)
Working, n (%)	137 (67.8)
Underlying disease, n (%)
Cardiovascular tract disease	48 (30.0)
Respiratory tract disease	26 (23.8)
Gastrointestinal tract disease	20 (12.5)
Cancer	21 (15.0)
Diabetes	25 (13.4)
Others	21 (26.3)
None	63 (37.0)
Number of times an ambulance was used, means (SD)	2.0 (1.5)
Time of day ambulance was used, n (%)
00:00–06:00	38 (18.8)
06:00–12:00	63 (31.2)
12:00–18:00	46 (22.8)
18:00–24:00	55 (27.2)

*Note: SD, standard deviation.

### 3.1. Selecting items

A total of 202 (96.2%) respondents had complete PECPSS data and constituted the population included in the factor analysis. This was considered to be a sufficient sample size, similar to previous studies using EFA [17],[33]. First, of the 36 question items, two items with mean scores >4.5 were deleted. There were 12 pairs with correlation coefficients exceeding 0.7. One of these pairs was deleted and 12 items were excluded. Next, a factor analysis using the maximum likelihood method was performed. For the EFA with Promax rotation, two items with factor loadings <0.35 were removed, and 20 items were finally selected (Table 2).

The EFA yielded 20 questions representing five domains: teamwork (two items), explanation and communication (seven items), physical treatment and psychological support (five items), quickness of transport (three items), and environment in the ambulance (three items).

**Table 2.** Prehospital emergency care patient satisfaction scale (PECPSS).

		Factor 1	Factor 2	Factor 3	Factor 4	Factor 5
Factor 1: Teamwork (two items)	The EMTs were efficient in their activities.	0.931	0.1	0.013	−0.108	0.1
	The teamwork among the EMTs appeared to be good.	0.877	−0.168	0.012	0.131	0.193
Factor 2: Explanation and communication (seven items)	The EMTs treated me with care, respect, and compassion.	0.112	0.86	0.052	−0.027	0.121
	The EMTs took great care of my privacy.	0.239	0.817	−0.087	0.148	0.147
	The EMTs gave me the opportunity to ask questions.	−0.136	0.767	0.195	0.082	0.009
	The EMTs answered my questions appropriately.	0.293	0.631	−0.17	0.207	0.121
	The EMTs gave me a clear explanation of my condition before arriving at the hospital.	−0.025	0.618	0.518	−0.216	0.138
	The information from the EMTs regarding my physical condition was reliable.	0.252	0.497	0.388	−0.23	0.055
	The EMTs explained to me which hospital I was to be transported to.	−0.11	0.459	0.1	0.012	−0.014
Factor 3: Physical treatment and psychological support (five items)	The EMTs understood my symptoms.	−0.03	0.007	0.794	0.249	0.018
	The EMTs alleviated my anxiety and concerns.	0.01	−0.039	0.786	0.284	0.009
	The EMTs calmly performed the procedure on me.	−0.108	−0.133	0.731	0.225	−0.119
	The EMTs appropriately took care of me.	0.107	0.066	0.682	0.045	0.425
	The EMTs responded to my symptoms.	0.043	0.096	0.599	0.229	−0.004
Factor 4: Quickness of transport (three items)	The EMTs transported me quickly to the hospital.	0.143	−0.066	0.239	0.665	0.058
	The wait for the EMTs to arrive was within the time I had anticipated.	−0.13	0.35	0.151	0.607	0.056
	The destination was quickly determined.	0.094	0.005	−0.02	0.517	−0.009
Factor 5: Environment in the ambulance (three items)	The bedding in the ambulance was clean.	−0.068	−0.189	−0.057	0.103	0.614
	The temperature inside the ambulance during transport was well taken care of.	0.11	−0.54	0.166	−0.062	0.538
	I felt safe during the ambulance transport.	−0.483	0.177	−0.046	0.026	0.374

### 3.2. Internal consistency reliability

The internal consistency reliability (Cronbach's alpha) of the four domains of the PECPSS-20 ranged from 0.82 to 0.94 (Table 3), corresponding to good internal reliability.

**Table 3.** Internal consistency reliability of the PECPSS-20 domains.

PECPSS-20 domain score	Mean (SD)	Cronbach alpha
Teamwork	4.4 (0.6)	0.82
Explanation and communication	4.2 (0.6)	0.94
Physical treatment and psychological support	4.2 (0.8)	0.91
Quickness of transport	4.0 (0.9)	0.86
Environment in the ambulance	4.3 (0.8)	0.88

*Note: PECPSS-20, the 20-item prehospital emergency care patient satisfaction scale; SD, standard deviation.

### 3.3. Construct validity

**Figure 1.**
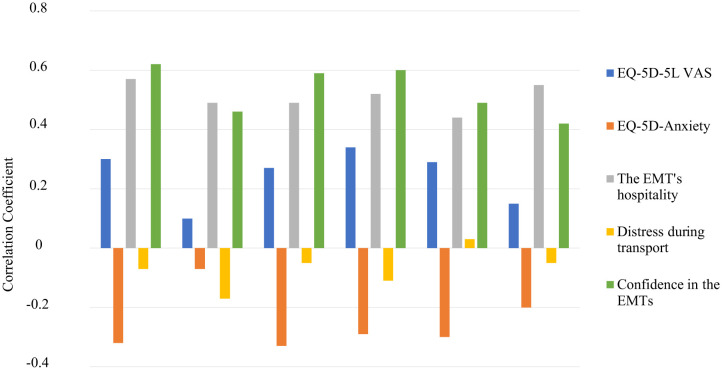
Construct validity of the 20-item prehospital emergency care patient satisfaction scale (PECPSS-20).

Correlation coefficients for the five items of the summary score and structural validity for each domain of the PECPSS-20 are shown in Figure 1. the EQ-5D-5L VAS was positively associated with the PECPSS-20 (0.30, p < 0.01). EQ-5D-5L Anxiety was negatively associated with PECPSS-20 (−0.32, p < 0.01), whereas EMTs' Hospitality and EMTs' confidence were positively associated with PECPSS-20 (0.57 P < 0.01), (0.62, p < 0.01) respectively. There was no correlation between distress during transport and the PECPSS-20 (−0.07, p = 0.52) (Table 4). All five construct validities we established were as hypothesized.

**Table 4.** Multifruit-multimethod correlations matrix.

Instrument	Score Mean (SD)		PECPSS-20 domain score				
		Summary Score	Factor 1	Factor 2	Factor 3	Factor 4	Factor 5
EQ-5D-5L VAS	62.1 ± 23.2	0.30	0.10	0.27	0.34	0.29	0.15
EQ-5D-Anxiety	1.9 ± 1.2	−0.32	−0.07	−0.33	−0.29	−0.30	−0.20
EMTs' hospitality	84.8 ± 23.5	0.57	0.49	0.49	0.52	0.44	0.55
Distress during transport	46.2 ± 37.4	−0.07	−0.17	−0.05	−0.11	0.03	−0.05
Confidence in the EMTs	85.2 ± 24.0	0.62	0.46	0.59	0.60	0.49	0.42

*Note: PCEPSS-20, the 20-item prehospital emergency care patient satisfaction scale; SD, standard deviation; VAS, visual analog scale; factor 1, Teamwork; factor 2, Explanation and communication; factor 3, Physical treatment and psychological support ; factor 4, Quickness of transport ; factor 5, Environment in the ambulance.

## 4. Discussion

In this study, we developed a patient satisfaction scale focused on EMTs and confirmed its validity and reliability. We propose that the scale can be used in prehospital settings, an important outcome of this study as to our knowledge, there are no reports on the development of patient satisfaction scales focused on EMTs. The results of the factor analysis revealed five key domains: “teamwork”, “explanation and communication”, “physical treatment and psychological support”, “promptness of transport”, and “environment in the ambulance”. This multidimensional structure is consistent with many studies that involved the development of a patient satisfaction scale [2],[34]–[36]. Thus, the five domains identified in this study have some similarities to the constructs of other scales [37],[38], which we consider supporting the construct validity of the current scale.

A limitation of measuring prehospital patient satisfaction is that the prehospital environment and scene factors are not adequately considered. Using existing patient satisfaction scales in emergency medicine may not effectively measure satisfaction with prehospital emergency care from the patient's perspective due to differences in prehospital settings [39]. Given the special situation wherein prehospital emergency care requires a more rapid response than in hospitals, a specific scale tailored to the phase of emergency care was considered essential, warranting a patient satisfaction scale that takes into account the prehospital settings.

The first domain of the PCEPSS-20 is “teamwork”. EMTs are expected to respond quickly to injured patients in a short period of time and with minimal medical resources [40]; the importance of teamwork among EMTs has been reported in other studies and is one of the most important factors in saving a patient's life [41]–[43]. Organizational teamwork has been reported to be associated with satisfaction, and EMTs need to function in the best interest of the patient [44]. Therefore, collaboration among EMTs to provide prehospital emergency care is considered integral.

The PCEPSS-20 domain “explanation and communication” included communication and the provision of information between EMTs and patients. Prehospital patients' needs include the desire for adequate explanations [45],[46]; EMTs can improve patient satisfaction by providing patients with explanations of situations and reliable information [12],[47]. In the uncertain prehospital setting, providing adequate explanations to the patient is an important factor in reducing patient anxiety and other concerns, and is considered to improve prehospital patient satisfaction [48].

The third domain is “physical treatment and psychological support”. Ambulance patients suffer from chronic pain [49] and diverse acute symptoms [50]. Previous studies have shown that symptom management and medical treatment are important for patients treated in the ambulance [51]–[53]. In addition, ambulance patients rate the technical skills and knowledge of EMTs highly [54], and these are considered important quality indicators for prehospital care. Moreover, patients who use ambulances are faced with unexpected situations and experience fear, anxiety, and apprehension [55]. EMTs need to be able to quickly allay anxiety through timely responses [56]. Specifically, physical care and psychological support are considered essential to provide care that aligns with the needs of the ambulance patient.

The fourth domain is “quickness of transport”. In a report assessing satisfaction with a large urban EMS system, respondents shared that for them, the most important part of the system was the quickness of transport [57]. Other reports have also shown a high level of patient dissatisfaction with transport delays [58]. Although each local system makes a different decision as to which hospital to immediately transport a patient to, evidently, quickness of transport is important from the patient's perspective and contributes to patient satisfaction.

The last domain is “environment in the ambulance”. A recent systematic review points to the importance of ambulance driving skills and comfort in the ambulance [59]. In patient satisfaction surveys in hospitals, the comfort and appearance of the facility and the availability of equipment are included among the domains [60],[61]. In the prehospital setting, EMTs perform various procedures in the ambulance. Ambulance care includes problems such as low ambient temperatures, and patients have reported negative experiences [62]. In addition, vibration, noise, temperature changes, limited space in the ambulance, and unexpected events affect the clinical condition of patients during interhospital transport [63]. We consider patient comfort and safety balanced against the potential benefits of rapid transport to be the key to patient satisfaction.

Cronbach's alpha for all domains of the PCEPSS-20 scale was greater than 0.8, indicating that the PCEPSS-20 exhibited similar values to previous patient satisfaction scales [3],[34] that have been validated for internal consistency. This means that each element showed adequate homogeneity.

All five items used for construct validity were as hypothesized. High patient QOL, high hospitality [51],[64],[65], and high trust [66],[67] have been reported to be associated with patient satisfaction, similar to the results of this study. High patient anxiety[68]–[70] has also been reported to be negatively associated with patient satisfaction, similar to previous reports and the present study. Furthermore, it has been reported that there is no correlation between the intensity of distress and patient satisfaction [71], which is consistent with our findings.

This study has several limitations. First, this study had a low response rate of 40.2%, which may have contributed to selection bias. However, other sociological surveys in the Hokkaido region typically have response rates between 30% [72],[73] and 50% [74], and the results of this study can be considered to be based on a representative sample of participants in this region. Second, we did not assess test-retest reliability. In research involving the development of psychometric instruments, test-retest reliability is essential to confirm the repeated administration of the same instrument [75]. This is an important area for future research to confirm the reliability of the PCEPSS-20. Third, concerns exist regarding the timeline of data collection. Data for this study were collected during the COVID-19 pandemic period. As a result, the prehospital emergency care system was likely different from normal, which could have affected the assessment of patients.

## 5. Conclusions

This study found the PECPSS-20 to be a robust measure of patient satisfaction, suggesting that it can measure satisfaction with EMTs. The PECPSS-20 is designed to focus on the activities of prehospital care EMTs, and the items reflect patient needs in prehospital care, determined based on an extensive literature search and content validity analysis. The PECPSS-20 consists of 20 items across five domains, showing high correlations with confidence in the EQ-5D and EMTs in construct validity. Measuring patient satisfaction with EMTs in prehospital care may help identify factors that are inadequate in the care services provided and guide measures to improve the quality of prehospital care.
